# Clinical Characteristics and Risk Factors of In-Hospital Mortality in Patients With Acute Myocardial Infarction With Subsequent Gastrointestinal Bleeding: A Single-Center Experience

**DOI:** 10.3389/fcvm.2022.942467

**Published:** 2022-07-13

**Authors:** Xin Su, Yuzhen Wei, Shuo Pang, Zeqing Zhang, Yunxiao Zhang, Peipei Zheng, Haiyu Li, Haiqiang Sang, Jianzeng Dong

**Affiliations:** ^1^Department of Cardiology, The First Affiliated Hospital of Zhengzhou University, Zhengzhou, China; ^2^Department of Cardiology, Beijing Anzhen Hospital, Capital Medical University, Beijing, China

**Keywords:** acute myocardial infarction, gastrointestinal bleeding, in-hospital mortality, thrombolysis, percutaneous coronary intervention

## Abstract

**Objective:**

Gastrointestinal bleeding (GIB) post acute myocardial infarction (AMI) is a severe clinical condition with a poor prognosis. The purpose of the study was to evaluate the rate of in-hospital mortality in patients with GIB post-AMI and to identify the potential risk factors of this situation.

**Methods:**

In this single-center retrospective study, a total of 154 patients diagnosed with AMI who subsequently suffered GIB were enrolled from October 2013 to December 2021. Demographic, laboratory, and clinical data were collected. The in-hospital mortality was the outcome of interest. Logistic regression analysis was used to investigate the potential risk factors of in-hospital mortality.

**Results:**

Among the 154 subjects included in the final analysis, the mean age was 65.58 ± 11.20 years, and 104 (67.53%) were males. GIB occurred in 11 patients after thrombolytic therapy, 50 patients after percutaneous coronary intervention (PCI), and 93 patients during drug conservative treatment. A total of 41 patients died in the hospital. The in-hospital mortality rate of the thrombolysis group, PCI group, and drug conservative treatment group was 27.27% (3/11), 28.00% (14/50), and 25.81% (24/93), respectively. There was no difference in the in-hospital mortality among the three groups. The multivariate logistic regression analysis showed that the peak levels of TnI (OR 1.07, 95% CI 1.02–1.12, *P* = 0.011), condition of cardiogenic shock after admission (OR 14.52, 95% CI 3.36–62.62, *P* < 0.001), and the use of the mechanical ventilator (OR 8.14, 95% CI 2.03–32.59, *P* = 0.003) were significantly associated with in-hospital mortality.

**Conclusion:**

Regardless of the treatment strategy for AMI, once GIB occurred, the prognosis was poor. High in-hospital mortality in patients with GIB post-AMI was independently associated with the peak levels of TnI, condition of cardiogenic shock, and the use of a mechanical ventilator.

## Introduction

Acute myocardial infarction (AMI) is a category of disease associated with the greatest mortality and morbidity (1, 2). Meanwhile, gastrointestinal bleeding (GIB) is a common medical condition that may lead to substantial morbidity and mortality (3–5). Once patients diagnosed with AMI subsequently suffer GIB, the situation will worsen.

Dual antiplatelet therapy (DAPT) with aspirin and a thienopyridine derivative is the common anti-platelet strategy after AMI irrespective of conservative or invasive treatment (6, 7). While the combined use of antithrombotic medications may reduce the risk of cardiovascular events, they increase the risk of hemorrhage events. GIB is a common cause of hemorrhage in AMI patients (8). Studies have reported GIB rates from 0.6 to 3.9% in AMI patients, which is associated with an increased risk of poor prognosis (9–13). A retrospective analysis of patients undergoing primary percutaneous coronary intervention (PCI) found that GIB was an independent predictor of all-cause mortality (14). Multiple factors, such as treatment contradiction and complicated conditions, may lead to adverse clinical outcomes for patients with GIB post-AMI.

At present, there are no guidelines to define the etiology, risk factors, and treatment principles in patients with GIB post-AMI. Considering the poor prognosis of patients with GIB post-AMI, it is critical to identify patients with an increased risk of in-hospital mortality, thereby increasing vigilance for these patients. Therefore, the purpose of the study was to evaluate the rate of in-hospital mortality in patients with GIB post-AMI and to identify the potential risk factors of this situation.

## Materials and Methods

### Study Population

This present study was a single-center retrospective analysis of hospitalized patients with AMI and GIB at the First Affiliated Hospital of Zhengzhou University, Henan, China. Patients admitted because of GIB post-AMI were retrospectively enrolled between October 2013 and December 2021. The inclusion criterion was confirmed admission diagnosis of AMI with subsequent GIB. Patients with a positive fecal occult blood test but no visible melena or without any other clinical evidence of GIB were excluded. In addition, subjects with GIB who subsequently suffered an AMI were also excluded.

According to the timing of GIB and different managements of AMI, we divided the AMI patients into three groups: thrombolysis group, PCI group, and drug conservative treatment group. The patients in the thrombolysis group had GIB after thrombolysis, patients in the PCI group suffered GIB after emergency PCI, and the drug conservative treatment group had GIB in simple antithrombotic therapy during the acute stage of AMI. This research was approved by the Ethics Committee of the First Affiliated Hospital of Zhengzhou University.

### Data Collection

We retrospectively collected data concerning patients’ demographic information, timing and main manifestations of GIB, types of AMI, comorbidities, medication history, admission features, physiological data, echocardiographic features, laboratory data, and clinical characteristics. Laboratory data were recorded at admission and rechecked (24, 48, 72 h, and then every 2–3 days) during hospitalization, including minimum values of hemoglobin, and peak values of creatine kinase muscle B (CK-MB), cardiac troponin I (TnI), and N-terminal pro-B-type natriuretic peptide (NT-pro BNP) for all patients. In this analysis, the markers of myocardial injury (CK-MB, TnI, and NT-pro BNP) were the peak values, and hemoglobin and red blood cells were the minimum values. Other laboratory data were based on admission data.

### Antithrombotic Therapy and Gastrointestinal Bleeding Treatment

In this study, patients diagnosed with AMI received a loading dose of aspirin (300 mg) plus clopidogrel (300 or 600 mg) or ticagrelor (180 mg), followed by a maintenance dose of aspirin (100 mg once a day) plus clopidogrel (75 mg once a day) or ticagrelor (90 mg twice a day). All patients with AMI in our center routinely used proton pump inhibitors (PPIs) to reduce the risk of GIB.

Once the diagnosis of GIB was clear, antithrombotic drugs would often be stopped, and the patients would increase the dosage of PPIs and use other mucosal protective drugs. Depending on the amount of blood loss and whether the bleeding continues, patients might require a blood transfusion. If possible, endoscopy is helpful in the treatment of GIB in patients with AMI. Meanwhile, in order to stop active GIB, some patients underwent interventional embolization. Once GIB is controlled, antithrombotic therapy will be resumed as soon as possible.

### Definition

Acute myocardial infarction was diagnosed according to the fourth universal definition (15), containing the ST-segment elevation AMI and the non-ST-segment elevation AMI. GIB was defined as clinically evident GIB (hematemesis, coffee-ground emesis and melena, and bloody stool) accompanied by decreased hemoglobin levels. Major GIB was defined as clinically evident GIB with a decrease in hemoglobin level of ≥2 g/dL from baseline (16–18). Each variable was defined in accordance with cardiovascular data standards (19). The primary outcome was in-hospital mortality from all causes, including cardiac death, multiple organ failure, massive hemorrhage/intracranial hemorrhage, and sudden death.

### Statistical Analysis

Categorical variables were shown as frequencies and percentages, whereas continuous variables were presented as mean ± standard deviations (SD). Continuous variables were compared using the one-way ANOVA analysis or Kruskal–Wallis test, whereas categorical variables were compared using the Chi-square test. Logistical regression was performed to evaluate the odds ratio (OR) and 95% confidence interval (CI) for the association between risk factors and in-hospital mortality. Variables significantly related to in-hospital mortality in univariate analysis (*P* < 0.05) and clinically relevant factors (age, sex, severity of GIB, and comorbidities) were input into one multiple logistic regression model to determine the risk factors of in-hospital mortality. The factors entered into the multivariate logistical regression analysis were as follows: age, sex, severity of GIB, hypertension, diabetes mellitus, coronary artery disease, previous stroke, admission heart rate, admission systolic blood pressure, left ventricular ejection fraction (LVEF), white blood cell, estimated glomerular filtration rate (eGFR), CK-MB, TnI, C-reactive protein (CRP), cardiogenic shock, mechanical ventilator, extracorporeal membrane oxygenation (ECMO), continuous renal replacement therapy (CRRT). In addition, cumulative incidence rates of in-hospital mortality were estimated by the Kaplan–Meier method and compared with the log-rank test. Statistical analyses were performed using SPSS 23.0 software. A two-sided *P*-value < 0.05 was considered statistically significant.

## Results

### General Characteristics of Study Patients

**FIGURE 1:**
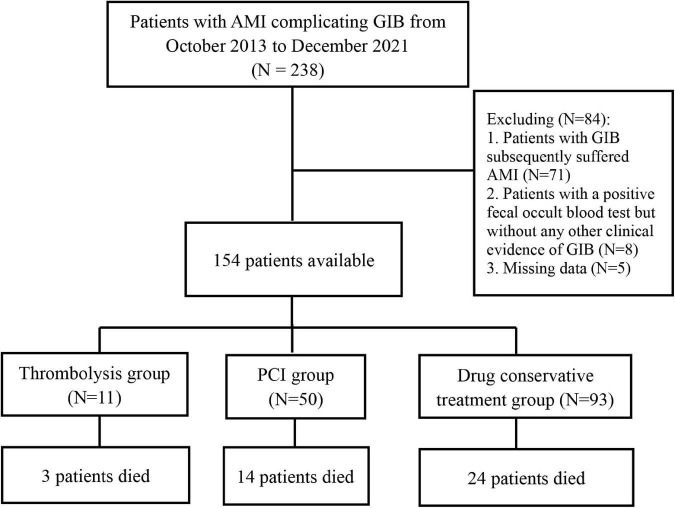
Flow chart for selection of study population. AMI, acute myocardial infarction; GIB, gastrointestinal bleeding; PCI, percutaneous coronary intervention.

Between October 2013 to December 2021, there were 238 patients diagnosed with GIB post-AMI in our center. A total of 154 patients were included after excluding 71 subjects diagnosed with GIB who subsequently suffered AMI, eight patients with a positive fecal occult blood test but without any other clinical evidence of GIB, and five patients with missing data (Figure 1).

Among the 154 subjects included in this study, 104 (67.53%) were males, and the mean age was 65.58 ± 11.20 years. Besides, GIB occurred in 11 patients after thrombolytic therapy (thrombolysis group), 50 patients after PCI (PCI group), and 93 patients during the drug conservative treatment (conservative treatment group).

Baseline characteristics according to the timing of GIB and different managements of AMI were illustrated in Table 1. Most characteristics had no difference among the three groups. The mean ages of the thrombolysis group, PCI group, and drug conservative group were 62.64 ± 10.95, 65.86 ± 11.31, and 65.78 ± 11.24, respectively. In addition, among the three groups, 9 (81.82%), 32 (64.00%), and 63 (67.74%) were males; 11 (100.00%), 33 (66.00%), and 38 (40.86%) were STEMI; 8 (72.73%), 36 (72%), and 73 (78.49%) had major GIB. The levels of hemoglobin and albumin in the PCI group were higher, and the proportion of patients using IABP and ECMO was higher in the PCI group.

**TABLE 1:** Characteristics of patients with GIB post-AMI stratified by different managements of AMI.

Demographic variables	Thrombolysis group (*n* = 11)	PCI group (*n* = 50)	Drug conservative treatment group (*n* = 93)	*P*-value
Age, years	62.64 ± 10.95	65.86 ± 11.31	65.78 ± 11.24	0.666
Male, *n* (%)	9 (81.82)	32 (64.00)	63 (67.74)	0.519
GIB to AMI time, days	3.27 ± 5.29	4.18 ± 5.12	2.59 ± 4.19	0.430
Type of AMI, *n* (%)				0.001
STEMI	11 (100.00)	33 (66.00)	38 (40.86)	
NSTEMI	0 (0.00)	17 (34.00)	55 (59.14)	
Main manifestation of GIB, *n* (%)				0.074
Hematemesis or coffee-ground emesis	5 (45.45)	17 (34.00)	19 (20.43)	
Melena or bloody stool	6 (54.55)	33 (66.00)	74 (79.57)	
Major GIB, *n* (%)	8 (72.73)	36 (72.00)	73 (78.49)	0.664
Medical history, *n* (%)				
Hypertension	5 (45.45)	31 (62.00)	46 (49.46)	0.310
Diabetes mellitus	2 (18.18)	15 (30.00)	41 (44.09)	0.097
Coronary artery disease	1 (9.09)	13 (26.00)	26 (27.96)	0.402
Previous stroke	3 (27.27)	12 (24.00)	14 (15.05)	0.324
Gastrointestinal disease	1 (9.09)	8 (16.00)	10 (10.75)	0.624
Current smoker, *n* (%)	4 (36.36)	15 (30.00)	21 (22.58)	0.450
Current drinker, *n* (%)	1 (9.09)	5 (10.00)	9 (9.68)	0.995
Medication history, *n* (%)				
Aspirin	0 (0.00)	7 (14.00)	14 (15.05)	0.387
P2Y12 receptor inhibitor	0 (0.00)	0 (0.00)	4 (4.30)	0.260
Admission features				
Admission heart rate, beats/min	88.18 ± 31.09	85.78 ± 25.71	86.25 ± 22.52	0.957
Admission systolic BP, mmHg	110 ± 22.49	118.84 ± 24.51	122.55 ± 25.24	0.247
Admission diastolic BP, mmHg	71.18 ± 15.01	72.42 ± 16.7	71.39 ± 15.17	0.926
LVEF, %	51.55 ± 8.63	44.6 ± 12.39	48.35 ± 10.09	0.061
Antithrombotic medications before GIB, *n* (%)				
Aspirin	11 (100.00)	50 (100.00)	93 (100.00)	–
Clopidogrel	7 (63.64)	23 (46.00)	62 (66.67)	0.054
Ticagrelor	4 (36.36)	27 (54.00)	31 (33.33)	0.054
Examinations				
Min hemoglobin, g/L	79.87 ± 19.3	83.07 ± 20.00	73.91 ± 19.86	0.031
Min red blood cell, 10^12^/L	2.8 ± 0.52	2.93 ± 0.63	2.67 ± 0.74	0.103
Platelet, 10^9^/L	219.09 ± 87.83	228.12 ± 89.7	229.28 ± 95.27	0.943
White blood cell, 10^9^/L	11.75 ± 3.84	12.04 ± 6.49	11.5 ± 6.48	0.889
Albumin, g/L	31.91 ± 5.09	36.6 ± 6.39	33.85 ± 5.94	0.012
Cr, μmol/L	94.46 ± 36.56	152.33 ± 176.05	166.46 ± 148.46	0.332
BUN, mmol/L	9.29 ± 4.34	9.89 ± 8.08	12.43 ± 8.87	0.159
eGFR, ml/min/1.73 m^2^	75.49 ± 25.05	62.1 ± 31.68	59.12 ± 33.37	0.280
Peak NT-proBNP, ng/L	8,198.45 ± 10,355.15	13,606.41 ± 22,967.4	15,731.51 ± 18,844.13	0.459
Peak CK-MB, μg/L	124.29 ± 254.9	180.27 ± 293.5	93.34 ± 174.11	0.125
Peak TnI, μg/L	15.11 ± 20.38	15.71 ± 22.43	9.74 ± 17.96	0.198
D-dimer, mg/L	1.11 ± 2.31	1.67 ± 3.97	1.68 ± 3.34	0.869
PT, s	12.44 ± 2.62	13.06 ± 6.68	12.37 ± 3.12	0.698
APTT, s	30.61 ± 5.37	34.89 ± 18.01	30.60 ± 5.36	0.451
TC, mmol/L	3.51 ± 1.02	3.63 ± 0.93	3.32 ± 1.04	0.219
TG, mmol/L	1.58 ± 0.79	1.22 ± 0.48	1.36 ± 0.7	0.500
LDL-C, mmol/L	1.98 ± 0.88	2.27 ± 0.85	1.94 ± 0.82	0.068
CRP, mg/L	42.4 ± 36.85	61.93 ± 69.38	43.82 ± 58.66	0.176
Clinical characteristics				
Blood transfusion, *n* (%)	6 (54.55)	27 (54.00)	61 (65.59)	0.359
Cardiogenic shock, *n* (%)	2 (18.18)	19 (38.00)	30 (32.26)	0.432
Mechanical ventilator, *n* (%)	5 (45.45)	15 (30.00)	28 (30.11)	0.569
IABP, *n* (%)	0 (0.00)	10 (20.00)	5 (5.38)	0.010
ECMO, *n* (%)	0 (0.00)	9 (18.00)	3 (3.23)	0.004
CRRT, *n* (%)	0 (0.00)	8 (16.00)	13 (13.98)	0.371
Length of hospital stay, days	13.18 ± 6.01	16.14 ± 13.53	14.94 ± 10.08	0.988
In-hospital mortality, *n* (%)	3 (27.27)	14 (28.00)	24 (25.81)	0.960

*AMI, acute myocardial infarction; GIB, gastrointestinal bleeding; PCI, percutaneous coronary intervention; STEMI, ST-segment elevation myocardial infarction; NSTEMI, non-ST-segment elevation myocardial infarction; BP, blood pressure; LVEF, left ventricular ejection fraction; Cr, creatinine; BUN, blood urea nitrogen; eGFR, estimated glomerular filtration rate; NT-proBNP, N-terminal pro-B-type natriuretic peptide; CK-MB, creatine kinase muscle B; TnI, troponin I; PT, prothrombin time; APTT, activated partial thromboplastin time; TC, total cholesterol; TG, triglycerides; LDL-C, low density lipoprotein cholesterol; CRP, C-reactive protein; IABP, intra-aortic balloon pump; ECMO, extracorporeal membrane oxygenation; CRRT, continuous renal replacement therapy.*

### Independent Risk Factors of In-Hospital Mortality

We conducted the regression analysis of factors associated with in-hospital mortality. Univariate logistic regression analysis revealed that admission heart rate (OR 1.02, 95% CI 1.01–1.04, *P* = 0.003), admission systolic blood pressure (OR 0.96, 95% CI 0.94–0.98, *P* < 0.001), LVEF (OR 0.91, 95% CI 0.87–0.94, *P* < 0.001), white blood cell (OR 1.11, 95% CI 1.04–1.18, *P* = 0.001), eGFR (OR 0.98, 95% CI 0.97–0.99, *P* = 0.017), CK-MB (OR 1.01, 95% CI 1.00–1.02, *P* = 0.001), TnI (OR 1.02, 95% CI 1.01–1.04, *P* = 0.001), CRP (OR 1.01, 95% CI 1.00–1.02, *P* = 0.048), cardiogenic shock (OR 27.42, 95% CI 10.46–71.86, *P* < 0.001), mechanical ventilator (OR 17.51, 95% CI 7.26–42.19, *P* < 0.001), ECMO (OR 17.90, 95% CI 3.72–86.01, *P* < 0.001), and CRRT (OR 3.77, 95% CI 1.46–9.74, *P* = 0.006) were predictors of the in-hospital mortality. In addition, the multivariate logistic regression analysis showed that the peak levels of TnI (OR 1.07, 95% CI 1.02–1.12, *P* = 0.011), condition of cardiogenic shock after admission (OR 14.52, 95% CI 3.36–62.62, *P* < 0.001), and the use of a mechanical ventilator (OR 8.14, 95% CI 2.03–32.59, *P* = 0.003) were significantly associated with in-hospital mortality (Tables 2, 3).

**TABLE 2:** Independent risk factors of in-hospital mortality.

	In-hospital mortality			
Predictors	Crude OR (95% CI)	*P*-value	Adjusted OR (95% CI)	*P*-value
Age, years	0.99 (0.96–1.02)	0.794	1.06 (0.98–1.15)	0.141
Male	1.05 (0.49–2.26)	0.903	1.46 (0.29–7.29)	0.644
Revascularization managements (conservative treatment as reference)			–	–
PCI	0.92 (0.22–3.78)	0.916	–	–
Thrombolysis	1.03 (0.24–4.48)	0.961	–	–
Type of AMI (NSTEMI as reference)				
STEMI	1.34 (0.65–2.76)	0.429	–	–
Main manifestation of GIB (hematemesis as reference)				
Melena or bloody stool	0.61 (0.28–1.32)	0.206	–	–
Major GIB	0.97 (0.42–2.24)	0.949	1.20 (0.22–6.52)	0.833
Hypertension	0.59 (0.29–1.23)	0.163	0.66 (0.15–2.88)	0.581
Diabetes mellitus	0.70 (0.32–1.49)	0.359	1.23 (0.20–7.47)	0.821
Coronary artery disease	0.39 (0.15–1.03)	0.059	0.19 (0.03–1.42)	0.108
Previous stroke	1.31 (0.54–3.16)	0.552	0.82 (0.16–4.16)	0.812
Gastrointestinal disease	0.29 (0.06–1.31)	0.108	–	–
Admission heart rate, beats/min	1.02 (1.01–1.04)	0.003	1.01 (0.99–1.04)	0.146
Admission systolic BP, mmHg	0.96 (0.94–0.98)	<0.001	0.98 (0.95–1.01)	0.210
LVEF, %	0.91 (0.87–0.94)	<0.001	0.94 (0.87–1.02)	0.135
Hemoglobin, g/L	0.99 (0.97–1.01)	0.698	–	–
White blood cell, 10^9^/L	1.11 (1.04–1.18)	0.001	1.03 (0.91–1.15)	0.670
Albumin, g/L	0.94 (0.88–1.01)	0.062	–	
eGFR, ml/min/1.73 m^2^	0.98 (0.97–0.99)	0.017	0.98 (0.95–1.01)	0.181
NT-proBNP, ng/L	1.00 (0.99–1.01)	0.424	–	–
CK-MB, μg/L	1.01 (1.00–1.02)	0.001	1.00 (0.99–1.01)	0.677
TnI, μg/L	1.02 (1.01–1.04)	0.001	1.07 (1.02–1.12)	0.011
D-dimer, mg/L	1.09 (0.99–1.21)	0.070	–	
CRP, mg/L	1.01 (1.00–1.02)	0.048	0.99 (0.98–1.01)	0.138
Blood transfusion	1.78 (0.82–3.85)	0.140	–	–
Cardiogenic shock	27.42 (10.46–71.86)	<0.001	14.52 (3.36–62.62)	<0.001
Mechanical ventilator	17.51 (7.26–42.19)	<0.001	8.14 (2.03–32.59)	0.003
IABP	1.98 (0.65–5.96)	0.224	–	–
ECMO	17.90 (3.72–86.01)	<0.001	4.82 (0.32–73.07)	0.257
CRRT	3.77 (1.46–9.74)	0.006	0.49 (0.06–4.32)	0.524

*OR, odds ratio; CI, confidence intervals; PCI, percutaneous coronary intervention; AMI, acute myocardial infarction; GIB, gastrointestinal bleeding; STEMI, ST-segment elevation myocardial infarction; NSTEMI, non-ST-segment elevation myocardial infarction; BP, blood pressure; LVEF, left ventricular ejection fraction; eGFR, estimated glomerular filtration rate; NT-proBNP, N-terminal pro-B-type natriuretic peptide; CK-MB, creatine kinase muscle B; TnI, troponin I; CRP, C-reactive protein; IABP, intra-aortic balloon pump; ECMO, extracorporeal membrane oxygenation; CRRT, continuous renal replacement therapy.*

**TABLE 3:** The multivariate logistic regression of in-hospital mortality with the beta coefficient and the confident interval.

Predictors	β (95% CI)	Adjusted OR (95% CI)	*P*-value
Age, years	0.06 (−0.06, 0.17)	1.06 (0.98, 1.15)	0.141
Male	0.37 (−0.36, 1.12)	1.46 (0.29, 7.29)	0.644
Major GIB	0.18 (−0.17, 0.53)	1.20 (0.22, 6.52)	0.833
Hypertension	−0.41 (−1.23, 0.39)	0.66 (0.15, 2.88)	0.581
Diabetes mellitus	0.21 (−0.20, 0.61)	1.23 (0.20, 7.47)	0.821
Coronary artery disease	−1.61 (−4.78, 1.55)	0.19 (0.03, 1.42)	0.108
Previous stroke	−0.19 (−0.58, 0.18)	0.82 (0.16, 4.16)	0.812
Admission heart rate, beats/min	0.01 (−0.01, 0.05)	1.01 (0.99, 1.04)	0.146
Admission systolic BP, mmHg	−0.02 (−0.06, 0.02)	0.98 (0.95, 1.01)	0.210
LVEF, %	−0.05 (−0.17, 0.05)	0.94 (0.87, 1.02)	0.135
White blood cell, 10^9^/L	0.02(−0.02, 0.07)	1.03 (0.91, 1.15)	0.670
eGFR, ml/min/1.73 m^2^	−0.01 (−0.05, 0.01)	0.98 (0.95, 1.01)	0.181
CK-MB, μg/L	0.01 (−0.01, 0.01)	1.00 (0.99, 1.01)	0.677
TnI, μg/L	0.06 (−0.06, 0.18)	1.07 (1.02, 1.12)	0.011
CRP, mg/L	−0.01 (−0.02, 0.01)	0.99 (0.98, 1.01)	0.138
Cardiogenic shock	2.67 (−2.56, 7.92)	14.52 (3.36, 62.62)	<0.001
Mechanical ventilator	2.09 (−2.01, 6.21)	8.14 (2.03, 32.59)	0.003
ECMO	1.57 (−1.51, 4.65)	4.82 (0.32, 73.07)	0.257
CRRT	−0.71 (−2.08, 0.67)	0.49 (0.06, 4.32)	0.524

*CI, confidence intervals; GIB, gastrointestinal bleeding; BP, blood pressure; LVEF, left ventricular ejection fraction; eGFR, estimated glomerular filtration rate; CK-MB, creatine kinase muscle B; TnI, troponin I; CRP, C-reactive protein; ECMO, extracorporeal membrane oxygenation; CRRT, continuous renal replacement therapy.*

### Cumulative In-Hospital Mortality Rates

A total of 41 (26.62%) patients died in the hospital. The in-hospital mortality rate of the thrombolysis group, PCI group, and drug conservative treatment group was 27.27% (3/11), 28.00% (14/50), and 25.81% (24/93), respectively. Causes of death included refractory heart failure (25, 60.98%), hemorrhage event (4, 9.75%), multiple system organ failure (10, 24.39%), and unknown reasons (2, 4.88%).

**FIGURE 2:**
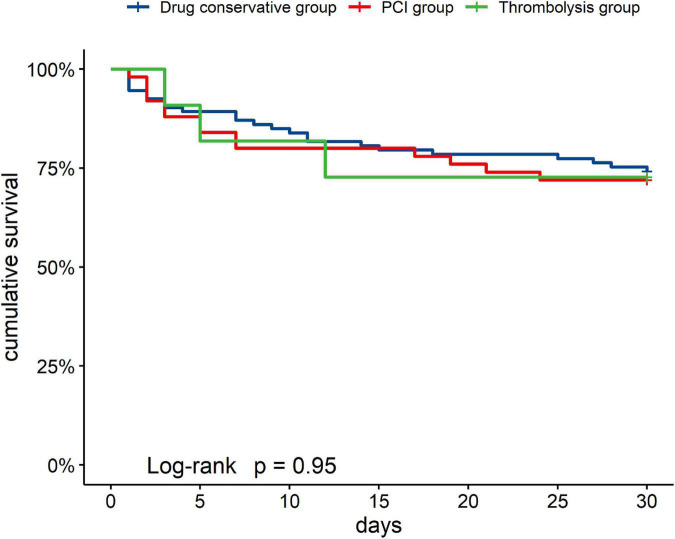
Kaplan–Meier estimates of cumulative event-free survival for in-hospital mortality.

Figure 2 showed the cumulative in-hospital survival rates of patients with different managements of AMI. There was no significant difference in the cumulative in-hospital mortality rates among the groups [thrombolysis group (27.27%, 3/11), PCI group (28.00%, 14/50), and drug conservative treatment group (25.81%, 24/93), Log-rank *P* = 0.950].

## Discussion

In this single-center retrospective study, we found that the in-hospital mortality of patients with AMI who subsequently suffered GIB was extremely high. In addition, regardless of the treatment strategy of AMI, once GIB occurred, the high in-hospital mortality was consistent. The peak levels of TnI, condition of cardiogenic shock, and the use of a mechanical ventilator were found to be independent predictors of poor prognosis.

To our knowledge, this study is one of the studies with the largest number of patients with GIB post-AMI, including individuals receiving invasive treatment strategies and non-invasive treatment strategies.

Dual antiplatelet therapy with aspirin and a thienopyridine derivative is the common antithrombotic strategy after AMI irrespective of conservative or invasive therapeutic methods (6, 7). This treatment reduces ischemic events but will be offset by increased bleeding events (20). Antiplatelet therapy will be limited once a bleeding event occurs. In addition, thrombolysis, as a treatment strategy for AMI, also has a high risk of bleeding. A multi-center study, data from the TIMI (Thrombolysis in Myocardial Infarction) trial, found that 0.4–1.8% of AMI patients receiving thrombolytic therapy suffered GIB events (21).

Gastrointestinal bleeding, including both lower and upper gastrointestinal origins, was independently associated with increased mortality in different conditions (11, 13, 14, 22). The ACUITY study illustrated that GIB was strongly associated with 30-day all-cause mortality [hazard ratio (HR) 4.87, interquartile range 2.61–9.08, *P* < 0.0001] (13). Sarajlic et al. demonstrated that upper GIB in AMI patients was associated with an increased risk of all cause-death (HR 2.86, 95% CI 2.583.1–6) and stroke (HR 1.80, 95% CI 1.32–2.45) (11). Meanwhile, Shalev et al. found that upper GIB in individuals with acute coronary syndrome (ACS) was associated with a markedly increased 30-day mortality (33%) (22).

In our study, the in-hospital mortality of AMI patients who subsequently suffered GIB was 26.62%. In one single-center retrospective study that included only patients diagnosed with non-ST-segment elevation AMI, the in-hospital mortality of individuals with GIB and AMI was 24.7% (23). Another retrospective study, data from the CAMI (China Acute Myocardial Infarction) Registry, noted that subjects with GIB had a significantly higher risk for death (HR 1.392; 95% CI 1.105–1.764, *P* = 0.0071), with an in-hospital mortality rate of 22% (10). In addition, Gaglia et al. identified GIB in 0.72% of 20,621 patients who underwent PCI, and the 30-day mortality rate of patients with GIB was 20.5% (24). Overall, our results are consistent and extend the evidence in patients with AMI who subsequently suffered GIB.

The mechanism of the poor prognosis in patients with GIB post-AMI may be multi-factorial. Firstly, GIB may lead to bleeding-related hemodynamic instability and aggravate ischemia, leading to stroke and reinfarction. Secondly, blood transfusion after GIB may have indirect effects, leading to systemic inflammation in the prethrombotic state, increasing oxidative stress, and paradoxically reducing oxygen delivery, all of which could lead to worse results (25). Besides, even mild bleeding without blood transfusion may result in the interruption of antithrombotic therapy, which could indirectly affect the prognosis (26). When antithrombotic therapy is suspended, the risk of acute stent thrombosis is extremely high, especially in patients undergoing primary PCI. Moreover, patients with GIB post-AMI had poor baseline clinical characteristics, such as older age and more comorbidities, which might be associated with poor outcomes. Based on the combined effect of the above reasons, the mortality of GIB post-AMI is higher than that of each situation.

The purpose of our study is to describe and explore potential predictors of in-hospital mortality in patients with GIB post-AMI. Univariate logistic regression analysis revealed that admission heart rate, admission systolic blood pressure, LVEF, white blood cell, eGFR, CK-MB, TnI, CRP, cardiogenic shock, mechanical ventilator, ECMO, and CRRT were predictors of the in-hospital mortality. Rapid heart rate and low blood pressure are the manifestations of cardiogenic shock. A retrospective study suggested that low systolic blood pressure and rapid heart rate at admission of STEMI patients were associated with a higher risk of in-hospital death (27). White blood cell counts and CRP as inflammatory markers in patients with AMI are independently associated with mortality (28, 29). Lower levels of eGFR and the use of CRRT mean that patients have acute or chronic kidney disease. Patients with chronic kidney disease experienced poor outcomes after AMI, while acute kidney injury was also associated with mortality in AMI patients (30, 31).

In the meantime, the multivariate logistic regression analysis showed that the peak levels of TnI, condition of cardiogenic shock, and the use of a mechanical ventilator were independent risk predictors of in-hospital mortality in AMI patients with GIB. To some extent, higher levels of TnI represent greater ranges of myocardial necrosis, which might be associated with poor prognosis. Widmer et al. found that TnI values ≥0.1 ng/ml were associated with higher in-patient mortality and 30-day readmission rates in myocardial injury patients (32). In addition, patients with cardiogenic shock often present with poor cardiac function and serious conditions, and these patients tend to have poor outcomes (33). A cardiogenic shock complicating AMI cohort study noted that the presence of respiratory failure and mechanical ventilator was associated with higher in-hospital mortality (34).

This research helps to identify patients with an increased risk of in-hospital mortality, thereby increasing vigilance for these patients. Patients with GIB post-AMI are in serious condition, and most of them are admitted to the intensive care unit for treatment. In the course of treatment, close monitoring should be carried out to prevent the complications of AMI and GIB.

Previous studies have found that factors such as previous bleeding, more intensive antithrombotic therapy, old age, and low hemoglobin are predictors of GIB in AMI patients (11, 12). Given the poor prognosis of GIB in individuals with AMI, it is more critical to prevent GIB in high-risk patients. PPIs or other mucosal protective agents could be commonly used in these patients to reduce the risk of GIB associated with antithrombotic therapy (8, 35). Besides, the intensity of antithrombotic therapy could be adjusted according to the risk of bleeding and ischemic event. Considering the adverse outcome of ischemic and hemorrhagic complication, the best treatment strategy must balance the risks of these events.

This clinical study has several limitations. Firstly, this was one single-center retrospective study, with the common shortcomings of analysis of the prerecorded data. Secondly, few patients underwent endoscopy in our study. Although some studies have pointed out that endoscopic treatment of GIB in patients with AMI is relatively safe (36–38), doctors usually choose relatively conservative treatment in order to avoid medical disputes. In addition, since we do not have the endoscopy results of these patients, the origin (lower and upper) of the GIB is unclear. Moreover, in our study, the severity of the disease and treatment methods are not consistent, but the treatment principles are consistent.

## Conclusion

The in-hospital mortality of AMI patients who subsequently suffered GIB was extremely high. Regardless of the treatment strategy of AMI, once GIB occurred, the prognosis was poor. The peak levels of TnI, condition of cardiogenic shock, and the use of a mechanical ventilator were found to be independent risk factors for poor prognosis.

## Data Availability Statement

The original contributions presented in this study are included in the article/supplementary material, further inquiries can be directed to the corresponding author.

## Ethics Statement

The studies involving human participants were reviewed and approved by the Human Research Ethics Committee of The First Affiliated Hospital of Zhengzhou University. The patients/participants provided their written informed consent to participate in this study.

## Author Contributions

XS, HS, and JD contributed to study planning and analysis, and were responsible for the overall content as guarantors. YW, SP, ZZ, YZ, PZ, and HL conducted the study and performed the examinations. XS and YW performed the statistical analysis and wrote the manuscript. All authors have read and approved the final version of the manuscript.

## Conflict of Interest

The authors declare that the research was conducted in the absence of any commercial or financial relationships that could be construed as a potential conflict of interest.

## Publisher’s Note

All claims expressed in this article are solely those of the authors and do not necessarily represent those of their affiliated organizations, or those of the publisher, the editors and the reviewers. Any product that may be evaluated in this article, or claim that may be made by its manufacturer, is not guaranteed or endorsed by the publisher.
